# Discontinuity in the Electronic Structure and Magnetic Order of *β*-Co_1+*x*_Ga_1−*x*_

**DOI:** 10.3390/ma15165523

**Published:** 2022-08-11

**Authors:** Gerhard H. Fecher

**Affiliations:** Max Planck Institute for Chemical Physics of Solids, D-01187 Dresden, Germany; fecher@cpfs.mpg.de

**Keywords:** CoGa, electronic structure, magnetism, binary alloys

## Abstract

The present work reports on the calculated electronic and magnetic structure of the binary Co-Ga system at high Co content. β-CoGa adopts a simple cubic CsCl type structure. Well-ordered CoGa does not exhibit collective magnetism but is a paramagnetic, metallic compound. Neither Co nor Ga deficiency induces magnetic order; however, ferromagnetism is observed for Co-Ga anti-site disorder. The magnetic moment per cell increases by up to approximately 1.2 $\mu_{B}$ in the completely disordered body-centered cubic structure. With increasing Co content, Co${}_{1+x}$Ga${}_{1-x}$ maintains the CsCl type structure and becomes ferromagnetic. Most importantly, a discontinuity of the magnetic order with composition is observed at about 10% excess Co, where a change from a low magnetic moment state to a high moment state is observed. This is accompanied by a change in the electronic structure and transport properties. The discontinuity is forced by the increasing exchange splitting related to the localized moment of the additional Co atoms that replace Ga. Subsequently, the magnetic moment increases continuously up to 2.5 $\mu_{B}$ for x=0.6. For x≳0.6, the structure changes to a face-centered cubic structure with random site occupation and the magnetic moment further increases. Above the magnetic discontinuity, the Curie temperature increases linearly with the Co content from the onset of ferromagnetism, until it reaches its maximum in pure Co.

## 1. Introduction

CoGa grown as thin films is of particular interest for spintronic applications. Suzuki et al. [1,2] reported on ultrathin MnGa films with perpendicular magnetic anisotropy that were grown on paramagnetic CoGa as a buffer layer. They reported on CoGa/MnGa/ MgO/CoFeB perpendicular magnetic tunnel junctions in reference [3] and in-plane, current-induced magnetization switching was observed in CoGa/MnGa/MgO films [4,5]. Such a current-induced, spin-orbit torque switching of the magnetization is of importance when utilizing perpendicularly magnetized magnetic tunnel junctions, which have been suggested for magnetic random access memory usage. Furthermore, CoGa was used as buffer layer to grow high-quality MnAl(001) films [6]. Lau et al. reported a high-spin Hall effect in paramagnetic CoGa thin films [7,8]. The use of CoGa (among others) for thin film and device growth by chemical templating was demonstrated by Filippou et al. [9].

Balster et al. used CoGa(001) as substrate to study the magnetic properties of ultrathin iron films [10]. Yasin et al. reported the magneto resistance for cluster spin glass Co-Ga alloys with slight (4–5%) Co excess [11,12]. Spin glass-type behaviour in Co-Ga alloys was reported already earlier by Meisel et al. for Co excess below 5% [13,14].

The phase diagram of the Co-Ga system was summarized in references [15,16], for example. In the present work, Co${}_{1+x}$Ga${}_{1-x}$ is investigated in the Co-rich part of its phase diagram with x≥1/2. A special emphasis is placed on the β phase. The β phase exhibits a simple cubic B2 crystal structure (prototype: CsCl, cP2, $P\;m\bar{3}m$ (221)) and exists in the composition range of about −0.2<x<0.4. Above 90% Co content, a face-centered cubic (fcc) lattice with A1 type structure (prototype: Cu, cF4, $F\;m\bar{3}m$ (225)) is observed, which is often assigned to α-Co. A mixture of the β and fcc phases occurs in the range between 70% and 90% Co content. At very high Co content above 98%, the hexagonal close packed (hcp) Co (ϵ-Co) structure that is the A3 type (prototype: Mg, hP2, $P\;6_{3}/mmc$ (194)) is observed. With the exception of pure Co and well-ordered CoGa, the crystal structures have random-site occupation in all alloys of the Co-Ga system considered here. It should be mentioned that the structure–composition relations change slightly between different reports [17,18,19,20].

The β-Co${}_{1+x}$Ga${}_{1-x}$ was experimentally investigated in the 1970s and 80s with respect to its magnetic [21,22,23,24] and transport [25] properties. Its electronic structure was rarely calculated and only reports on stoichiometric, paramagnetic CoGa are available [26,27].

It is interesting to note that β-CoGa is principally a parent material for the Co${}_{2}T$Ga Heusler compounds [28,29] where T= Ti, V, Cr, Mn, Fe, Nb, Hf [24,30], or others. Those Heusler compounds are all based on a CsCl 2×2×2 superstructure, where the Ga atoms are replaced by the *T* element in every second CsCl cell. That is, one has a transformation from Co${}_{2}$Ga${}_{2}$ to Co${}_{2}T$Ga. In many cases, Heusler compounds exhibit a B2-type disorder where one has Co($T_{1/2}$Ga${}_{1/2}$). In the present case, this corresponds to β-Co${}_{1+x}$Ga${}_{1-x}$ with x=1/2. In particular, the compounds where *T* is a 3d transition metal attracted a lot of attention in solid-state physics because they are suggested to be so-called half-metallic ferromagnets [31,32].

The present work investigates the electronic and magnetic structure of the alloys in the Co-rich part of the Co-Ga phase diagram with a special emphasis on β-Co${}_{1+x}$Ga${}_{1-x}$. Full potential, first-principles methods have been used to study well-ordered and disordered CoGa as well as varying composition with random-site occupation in the Co-Ga system.

## 2. Results and Discussion

### 2.1. Stoichiometric β-CoGa

**Figure 1:**
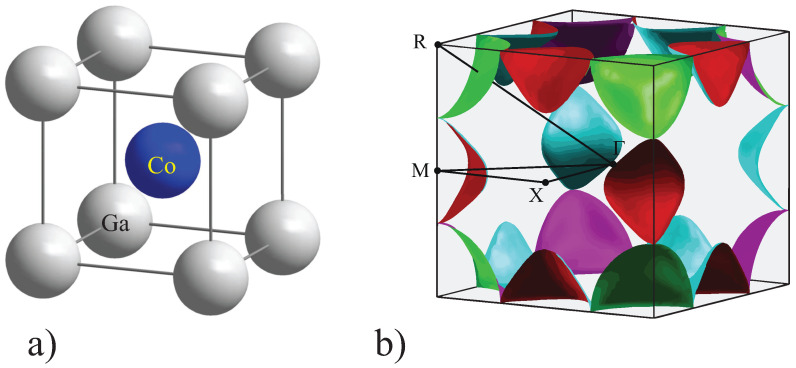
Real and reciprocal space structure of β-CoGa. (**a**) In the real space structure, the Ga atoms are located at (0, 0, 0) and the Co atoms at (1/2, 1/2, 1/2). (**b**) The Fermi surface of paramagnetic β-CoGa is plotted in the first Brillouin zone. The irreducible wedge of the first Brillouin zone is marked together with the points of high symmetry (Γ, X, M, R).

As a starting point, calculations were performed for stoichiometric, well-ordered β-CoGa. The real and reciprocal space structures are shown in Figure 1. The calculations were initialized for different magnetic states, defined by different starting spin configurations. Independent of the initial magnetic state, the resulting, converged ground state was always non-spin polarized.

**Figure 2:**
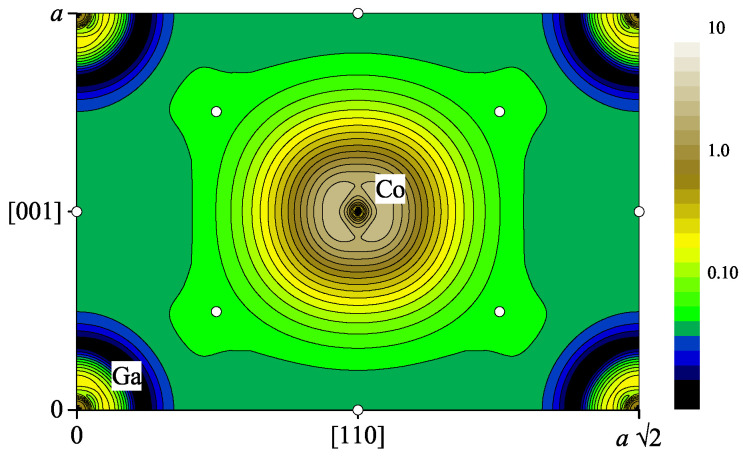
Charge density ρ(r) of paramagnetic β-CoGa. Shown is the valence charge density in the (110) plane. The bond-critical points are marked by open symbols (∘). (The $\log_{10}$ colour scale is in atomic units.)

The calculated valence charge density of CoGa is shown in Figure 2 for the (110) plane. The Co atom is located at the center of the figure. The minima of the valence charge densities are found close to the Ga atoms.

A more detailed analysis of the total charge density including the core level was performed by using Bader’s [33] quantum theory of atoms in molecules and solids (QTAIMS) analysis, based on Critic2 [34,35]. The topology of the charge density ρ(r) is characterized by critical points where ρ(r) has an extremal value, that is for ∇ρ(r)=0. Overall, eight different critical points were found as summarized in Table 1. These are the two nuclei, three bonds, two rings, and one cage-critical point. Naturally, the electron distribution has local maxima at the nuclei. The electron density is expected to be high in this region due to the tightly bounded core electrons. Two of the bond-critical points are located halfway between the Co or Ga atoms, whereas the third one is found along the [111] axis between Co and Ga, slightly closer to Ga. The 12-fold degenerate cage-critical point, that is the absolute minimum of the total charge density, is found close to the Ga atoms. It should be noted that the cage-critical point is not located in the (110) plane drawn in Figure 2.

The number of electrons found in the basins around the Co and Ga atoms are $Z_{\mathrm{Co}}=27.335$ and $Z_{\mathrm{Ga}}=30.665$, respectively. This difference corresponds to a charge transfer of approximately 0.3 electrons from Ga to Co. This is very similar to the situation in the Heusler compound Co${}_{2}$MnSi, where about 0.3 electrons are also transferred to each Co atom [36]. Flatness is a measure of metallicity [37]. Metallic systems exhibit a flat electron density ρ throughout the valence region. The flatness is defined by $f=\rho_{\min}^{c}/\rho_{\max}^{b}$ where $\rho_{\min}^{c}$ is the charge density at the cage-critical point and $\rho_{\max}^{b}$ is the highest density among all the bond critical points. From the QTAIMS analysis, it was determined that $f_{\mathrm{CoGa}}=0.671$ for β-CoGa. This indicated an even higher flatness compared to Cu ($f_{\mathrm{Cu}}\approx 0.57$ [37]). Based on the large flatness which is greater than that of other covalent compounds by a factor of 6 to 7, it can be concluded that the bonding in CoGa is clearly metallic, as expected.

Calculation of the lattice or Madelung energies [38] ($E_{M}$) is a fast way to verify the crystal stability [39,40]. For the present case of CoGa, one may compare the three most simple cubic structures for binaries, which are B1 (NaCl), B2 (CsCl), and B3 (ZnS). The dimensionless Madelung constants for these three structures are $c_{M}^{B1}=1.747564$, $c_{M}^{B2}=1.762675$, and $c_{M}^{B3}=1.633806$. The lattice energies are then found from

(1) $$E_{M}=-f\times c_{M}\times q^{2}/d_{NN},$$

where f=2778 kJ Å/mol. $d_{NN}$ is the nearest-neighbour distance in the equilibrium structure, and *q* is the effective charge from the Bader’s QTAIM analysis. $d_{NN}$ and *q* were calculated for all three structures. The obtained value for CoGa in the B2 structure is $E_{B2}=-220.75$ kJ/mol. The values for the other two structures are $E_{B1}/E_{B2}=0.272$ and $E_{B3}/E_{B2}=0.367$. From $E_{B2}<E_{B3}<E_{B1}$, the B2 structure is the most stable. Together with the formation enthalpy of ∆H=−50.3 kJ/mol (see Appendix A), this confirms the high stability of CoGa in the CsCl structure.

**Figure 3:**
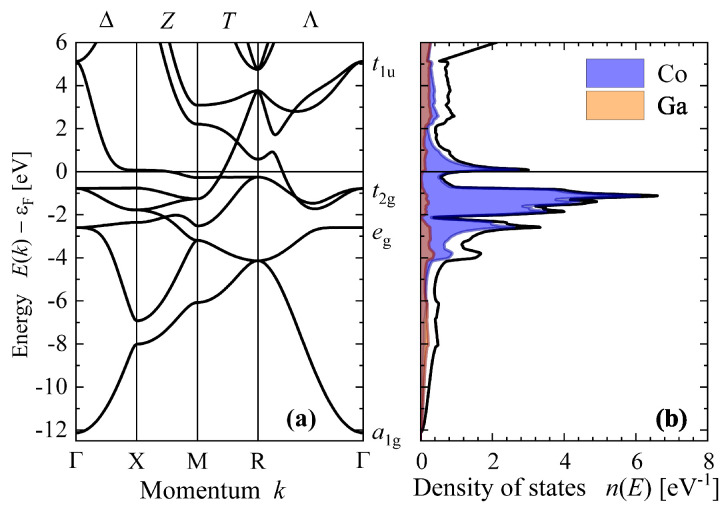
Electronic structure of paramagnetic β-CoGa. Shown are the band structure (**a**) and density of states (**b**). The irreducible representations of the states at Γ are marked. Notations of the high symmetry directions and points are given as top and bottom labels, respectively. Energies are given relative to the Fermi energy ($\epsilon_{F}$).

More details of the calculated electronic structure—band structure E(k) and density of states n(E)—is shown in Figure 3. The density of states as well as the shape of the dispersion curves are similar to the calculations presented by Whittle et al. [26]; however, the position of the Fermi energy is slightly shifted.

Bands are crossing the Fermi energy in the $Z=\bar{XM}$, $T=\bar{MR}$, and $\Lambda =\bar{R\Gamma }$ directions giving rise to pockets around the *M* and *R* points. These pockets are touching in the *T* direction through a common band crossing the Fermi energy. The missing crossing in the $∆=\bar{\Gamma X}$ direction hints that a closed energy surface is not formed around the Γ point.

Figure 1b shows the Fermi surface of paramagnetic CoGa. It consists of 8 pockets around the *R* points and 12 pockets around the *M* points, as previously mentioned in the discussion of the band structure. The pockets at *M* and *R* were characterized in reference [26] as electron and hole type, respectively. In the present work, the shape and position of the conduction band at *R* and the crossing of the Fermi energy by this band are different. The curvature of the Fermi surface is more detailed, and both negative and positive effective masses occur, depending on the direction in *k*-space. Therefore, the distinction between holes and electrons becomes ambiguous, and is omitted in subsequent discussions.

From the density of states, it is interesting to note that the states localized at the Ga atoms are nearly vanishing at the Fermi energy. Therefore, electronic transport might mainly be carried by the states that are localized at the Co atoms. Most interesting is the behavior of the band crossing the Fermi energy in the Λ direction corresponding to [111]. The topmost occupied state at the centre of the Brillouin zone has $\Gamma_{5g}$ ($t_{2g}$) character and splits into $\Gamma_{7+}$ ($e_{5/2g}$) and $\Gamma_{8+}$ ($f_{3/2g}$) when spin orbit interaction is included (see reference [41] for the irreducible representations). A further splitting of the bands is observed along the Λ direction where the valence state just below $\epsilon_{F}$ has $R_{8+}$ ($f_{3/2g}$) character whereas the character of the conduction state just above $\epsilon_{F}$ is $R_{7-}$ ($f_{5/2u}$). Only the $\Lambda_{5+6}$ ($e_{3/2}$) band is crossing the Fermi energy. It is interesting to note that this situation is similar to Pt, where also only the $\Lambda_{5+6}$ ($e_{3/2}$) band is crossing $\epsilon_{F}$ in the [111] direction. In that case, the initial $\Gamma_{8+}$ ($f_{3/2g}$) valence state becomes thereby split at Λ into a $e_{1/2}$ valence and a $e_{3/2}$ conduction state.

### 2.2. β-CoGa with Vacancies or Ga Excess

The β structure is stable starting from Co${}_{0.8}$Ga${}_{1.2}$, that is for Ga excess. Calculations for the Ga-rich part of the Co-Ga phase diagram resulted in a non-spin-polarized phase when all sites are fully occupied by assuming that the excess Ga replaces missing Co atoms on the 1b site resulting in (Co${}_{0.8}$Ga${}_{0.2}$)Ga. Alternatively, a structure with missing Co atoms might be assumed. In that case, one has a lattice build from a filled Ga cage with missing Co atoms in the center of the cubes and the compound seems to have a structure with a high amount of Schottky defects. A similar effect might appear for Co excess.

**Figure 4:**
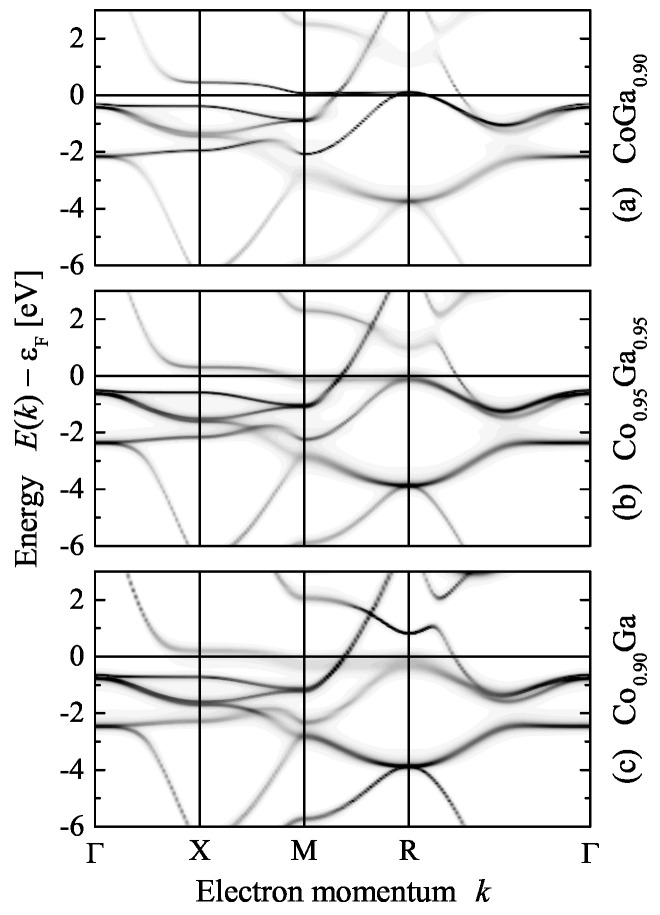
Electronic structure of CoGa with vacancies. Shown are the Bloch spectral functions at a total of 10% vacancies for three different distributions of Co and Ga as indicated: (**a**) Ga deficiency, (**b**) deficiency of both, and (**c**) Co deficiency.

Schottky defects may also appear for stoichiometric alloys at finite temperatures. In that case, one or more atoms are removed from their sites, leaving vacancies behind. In order to maintain the 1:1 composition, the same amount of Co and Ga were removed from the lattice sites. This resulted in (Co${}_{1-y}$${□}_{y}$)(Ga${}_{1-y}$${□}_{y}$), where □ is the Schottky symbol for the vacancies. The composition is changed when only one kind of atom (or a different amount) is removed from the lattice. If *y* Ga vacancies appear, that is for CoGa${}_{1-y}$—or in the more appropriate notation Co(Ga${}_{1-y}$${□}_{y}$)—the resulting composition is 1:(1−y) and similar (1−y):1 for Co vacancies. Calculations were performed for *y* up to 0.1. The impact of the vacancies on the electronic structure is demonstrated in Figure 4. The high value of the defect concentration was chosen to better visualize the effects on the electronic structure. The qualitative conclusions, however, also hold for much lower defect concentrations.

A small shift of the Fermi energy was observed due to the different amount of valence electrons when changing the composition. Furthermore, it is obvious that the removal of Co results in a broadening of the states close to the Fermi energy. This is in agreement with the fact that these states are localized at the Co atoms. The Co defect induced broadening of the bands carrying the transport causes an increase of the resistivity. In the case of Ga vacancies, a Co excess of about 5% is observed and the flat band between *M* and *R* is shifted slightly above the Fermi energy. No magnetic order was found for any of the three types of vacancies. The important implication of these observations is that an excess of Co produced by Ga vacancies results in a non-spin-polarized ground state.

### 2.3. Anti-Site Disordered CoGa

This section also deals with structural defects in stoichiometric CoGa and in particular, with anti-site disorder. In this case, Co and Ga atoms partially change their sites until a body-centered cubic A2 type structure (W, cI2, $I\;m\bar{3}m$ (229)) is formed where Co and Ga are randomly distributed on the doubly degenerate 2a lattice sites. Unlike the vacancies, the anti-site disorder induces ferromagnetic order.

For the electronic and magnetic structures, it was assumed that the two sites of the simple cubic CsCl lattice are occupied such that it corresponds to 1−z times the occupation in the B2 structure and *z* times that of the A2 structure. That means the 1b site occupation is 1−z/2 for Co and simultaneously z/2 for Ga; and accordingly, opposite for the 1a site. At 0≤z<1, the structure is still of the B2 type whereas it becomes A2 at z=1.

**Figure 5:**
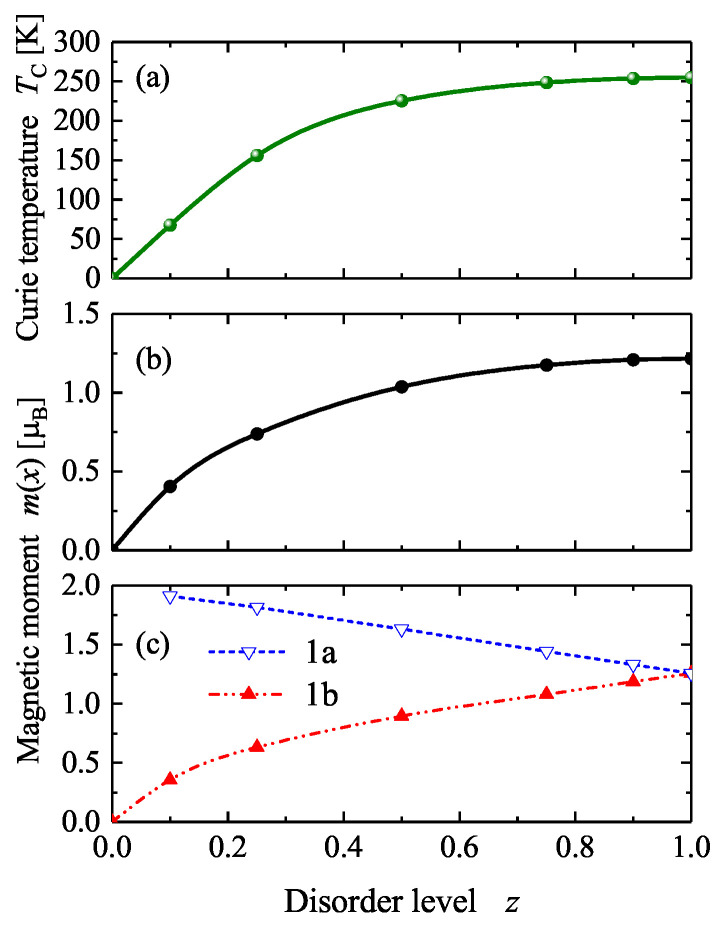
Magnetic properties of disordered CoGa. (**a**) The Curie temperature, (**b**) The total magnetic moment per primitive cell, and (**c**) The site-specific moments per atom (1a and 1b are the sites with low and high Co content, respectively).

The magnetic properties of the disordered CoGa are summarized in Figure 5. The total magnetic moment in the primitive cell increased non-linearly from zero to about 1.2 $\mu_{B}$. This is a different behavior than that of a mixture of two different compounds with B2 and A2 type structures. In that case, the magnetic moment would increase linearly from zero to the maximum at a 1:1 mixture. The non-linear behavior is attributable to the different magnetic moments of the Co atoms on the two non-equivalent sites. The moment at the Co replacing Ga decreases linearly whereas the moment at the Co atoms on the initial site increases non-linearly until both are finally equivalent in the A2 type structure. Furthermore, it was found that the Curie temperature increased with disorder in a non-linear manner, similar to the total magnetic moment. Its value reached a maximum of approximately 250 K in the completely disordered A2 structure. However, an analysis of the exchange coupling constants revealed that the spin stiffness was nearly constant with a value of about (225 ± 10) meVÅ${}^{2}$.

**Figure 6:**
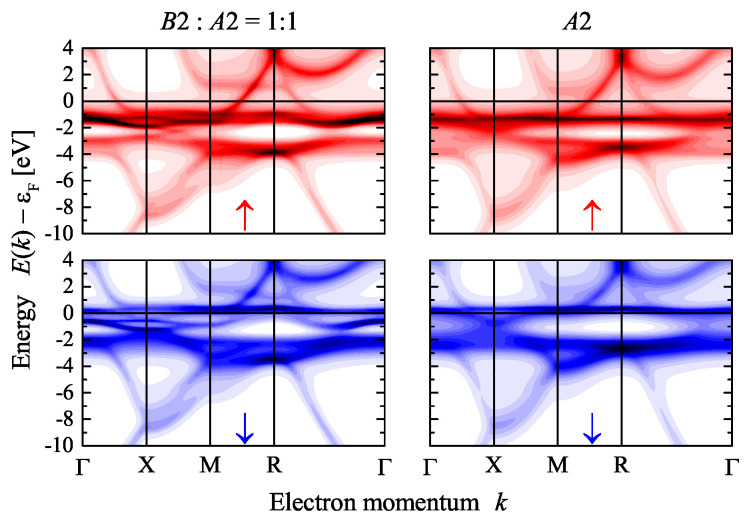
Electronic structure of disordered CoGa. Shown are the spin-resolved Bloch spectral functions of the disordered structures for z=0.5 (B2:A2=1:1) and z=1 (A2). The arrows ↑ and ↓ assign majority and minority states in the upper and lower row, respectively.

Figure 6 compares the Bloch spectral functions of disordered CoGa for the B2 structure with z=0.5 and the A2 structure. The electronic structure is similar in both cases; however, the broadening of the states by chemical disorder scattering is different. The magnetic exchange in the disordered CoGa splits the initial band structure of the ordered compound (see Figure 3) into majority and minority states. The exchange splitting shifts the majority states arising from the flat band between *X* and *M* below the Fermi energy. As a side effect, a majority band crossed the ∆ direction between Γ and *X*. This band changes the transport properties, in particular, for majority electrons. The minority Bloch spectral function exhibits flat bands just above the Fermi energy.

In concluding this section, it should be mentioned that the sensitivity of the magnetic order on the amount of anti-site disorder facilitates easy detection. Any small magnetic moment in an otherwise stoichiometric CoGa sample will provide an indication of this type of disorder.

### 2.4. β-CoGa with Co Excess (Co${}_{1+x}$Ga${}_{1-x}$, x<0.6)

Changes of the composition may have a wide range of consequences. The two sites of the B2 structure may either be fully occupied or vacancies may appear on one of the sites. The effect of vacancies was already discussed in Section 2.2, where it was shown that they do not lead to magnetic order. If *x* is the deviation from the 1:1 stoichiometry, then the first type results in the Co:Ga composition (1+x):(1−x). In this situation, the excess Co atoms occupy the Ga site, which might be expressed as Co(Co${}_{x}$Ga${}_{1-x}$). Unlike the situation where vacancies are present, the aforementioned case results in two non-equivalent Co atoms which leads to ferromagnetic order at high enough Co content.

**Figure 7:**
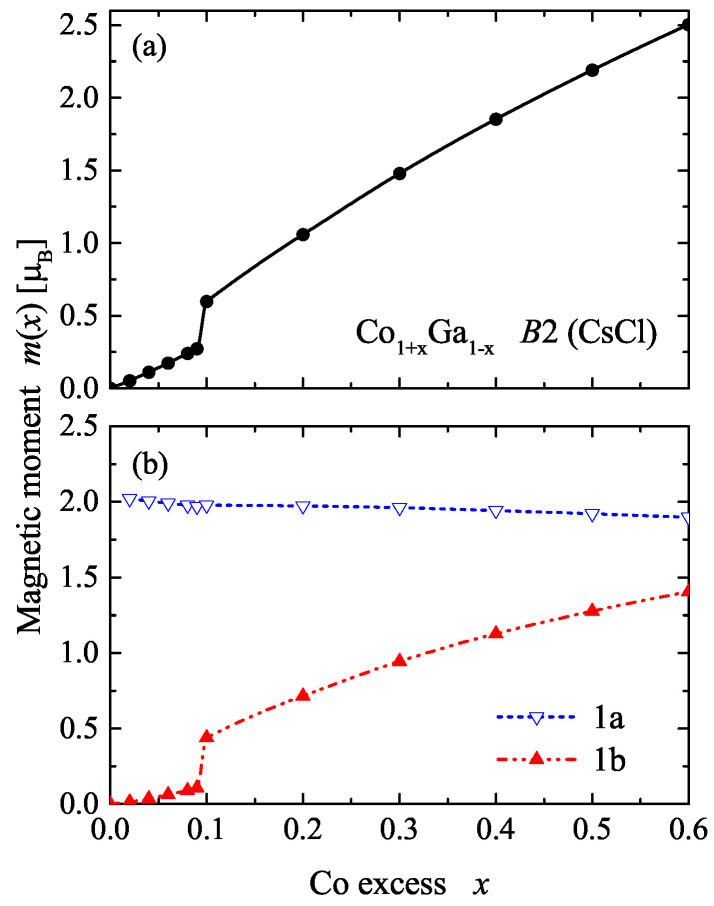
Magnetic moments of β-Co${}_{1+x}$Ga${}_{1-x}$. (**a**) The total magnetic moment per primitive cell. (**b**) The site-specific moments per atom (1a and 1b are the sites with low and high Co content, respectively).

Already for very low Co excess (x<0.05), the calculations converged into a spin-polarized ground state. However, the magnetic moment remains very low. This point will be further discussed in Section 2.5 together with the Curie temperatures. At x=0.06, a small magnetic moment is obtained and a sudden increase of the total magnetic moment is observed at x=0.1. The development of the total magnetic moment per primitive cell as well as the site-specific magnetic moments per atom is shown in Figure 7. The magnetic moment of Co in the original 1b position (1/2, 1/2, 1/2) increased from zero to approximately 1.5 $\mu_{B}$ at x=0.6 with a discontinuity at x=0.1. This is a different behavior from the case in which additional Co atoms replace Ga atoms on the 1a site (0, 0, 0). The moment of those Co atoms had a nearly constant value of about 2 $\mu_{B}$. The total magnetic moment per primitive cell increases accordingly from zero to 2.5 $\mu_{B}$.

**Figure 8:**
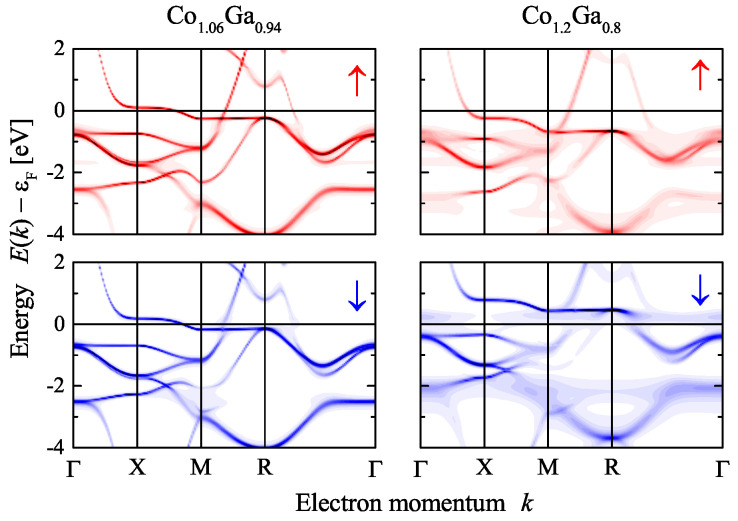
Electronic structure of β-Co${}_{1+x}$Ga${}_{1-x}$. Shown are the spin-resolved Bloch spectral functions for x=0.06 (**left**) and x=0.2 (**right**). The arrows ↑ and ↓ in the upper and lower row assign majority and minority states, respectively.

The changes of the electronic structure with composition are demonstrated in Figure 8. The Bloch spectral functions for Co excess of 6% and 20% are compared. At low Co excess where the onset of ferromagnetism occurs, only a small exchange splitting and the beginning of the broadening of the states by chemical disorder scattering is observed. It should be noted that the effect of disorder scattering for excess of Co is much less than for the anti-site disorder reported in Section 2.3. The bands crossing the Fermi energy are still similar to the band structure of the compound with 1:1 stoichiometry. In particular, the conduction bands in the ∆ direction are still above the Fermi energy for both spin states, minority and majority. The Fermi surfaces for these states are still similar and no major differences in the electronic transport properties were effected. The main changes in the conductivity are expected to be caused by the chemical disorder scattering.

The situation changes at higher Co content where a much larger magnetic exchange splitting is observed. In addition, the broadening of the states by chemical disorder scattering is more pronounced. At 20% Co excess, the majority conduction band crosses the Fermi energy in the ∆ direction. Furthermore, the minority band that initially crossed the Fermi energy between *M* and *R* is now shifted completely above it. The differences in the electronic structure shown for the case of the Bloch spectral function in Figure 8 explain the discontinuity of the magnetic moments. The number of valence electrons increases with Co content. Here it is assumed that for Co 9 and for Ga 3 electrons per atom contribute to the valence electron concentration. An increase in the valence electron concentration leads to an increase of the exchange splitting of the states. At a certain point, the flat majority band at *X* along ∆ and/or the flat minority band between *M* and *R* will touch the Fermi energy. This leads to an instability. At low Co excess (below 10%), the system is stable in the low moment state where both bands are above/below the Fermi energy. Above x=0.1, the enhanced exchange splitting—forced by the localized moment of the additional Co atoms in the 1a position—causes the system to turn into the high moment state.

**Figure 9:**
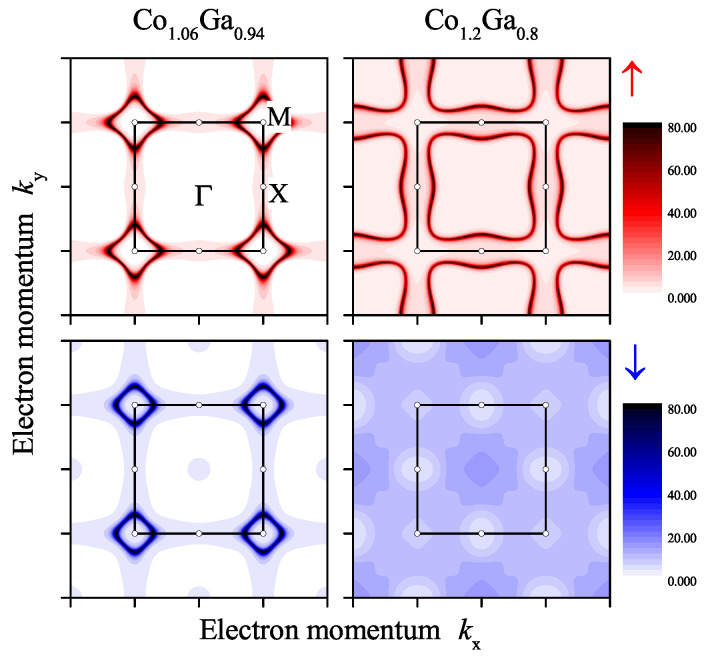
Fermi surface of β-Co${}_{1+x}$Ga${}_{1-x}$. Shown are spin-resolved Bloch spectral functions in the (001) plane of the Fermi surface for x=0.06 (**left**) and x=0.2 (**right**). The arrows ↑ (**upper row**) and ↓ (**lower row**) assign majority and minority states, respectively.

The different behavior of the states at the Fermi energy for Co excess below or above approximately 10%, leads to completely different Fermi surfaces for majority and minority electrons. As such, large differences in their transport properties are observed. The differences in the Fermi surfaces are demonstrated in Figure 9 for a low magnetic moment (x=0.06) and a high moment (x=0.2) state. The figure shows cuts through the majority and minority Fermi surfaces in the (001) plane. It was observed that the Fermi surface of the low magnetic moment state is similar to the paramagnetic state. However, the pockets at *M* are slightly smaller for minority compared to majority states. The Fermi surface has a completely different shape in the high magnetic moment state as expected from the dispersion results shown in Figure 8. In the high moment state, the bands are no longer crossing the Fermi energy in the *Z* direction between *X* and *M*. This involves both, majority and minority, electrons.

**Figure 10:**
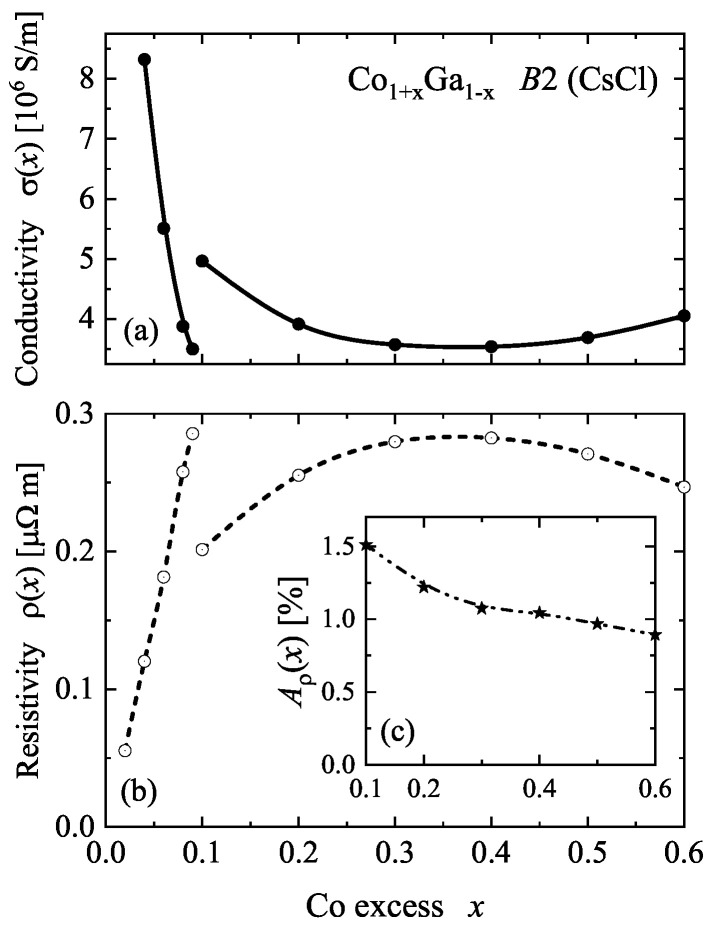
Electric transport of β-Co${}_{1+x}$Ga${}_{1-x}$. Shown are the conductivity (**a**), top and resistivity (**b**), bottom as function of the Co excess. The values are averages for current parallel or perpendicular to the magnetization. The inset (**c**) shows the magnetic transport asymmetry $A_{\rho }$.

The electric transport properties are shown in Figure 10. The resistivity exhibits a strong increase in the low magnetic moment range where x<0.1. A sudden discontinuity appears when the system changes into the high moment state. For higher Co excess, the resistivity exhibits a maximum at x≈0.375. It should be noted that the calculation was performed for 0 K and does not include scattering by phonons. The strong initial increase is caused by the chemical disorder scattering of electrons at the Co atoms that randomly replace Ga atoms. A sudden change of the conductivity at x=0.1 emerged from the change of the Fermi surface when the state changed from low to high magnetic moment. The maximum in the resistivity is typical for alloy systems [42]. It appears when the maximum of the chemical disorder scattering is achieved. Its position depends on the differences in the electron scattering from the atomic species of Co and Ga.

The magnetic transport asymmetry $A_{\rho }$ is defined by the difference of the resistivities parallel ($\rho_{\|}$) and perpendicular ($\rho_{⊥}$) to the magnetization. This value is calculated from $A_{\rho }=\left(\rho_{\|}-\rho_{⊥}\right)/\bar{\rho }$. The average resistivity $\bar{\rho }=tr\left(\rho \right)/3$ is calculated from the trace of the resistivity matrix ρ. The result for the high moment state is shown in the inset of Figure 10. It can be inferred that the positive sign indicates that the resistivity is higher when the current is parallel to the magnetization of the sample. The asymmetry is in the order of (1…1.5)% and decreases slightly with increasing Co content. $A_{\rho }\left(x\right)$ is in the same order of magnitude for x<0.1; however, the calculated values are very noisy due to the low magnetic moments. Therefore, they are omitted from this analysis.

### 2.5. From Half to Full Co Content: From CoGa to Co

**Figure 11:**
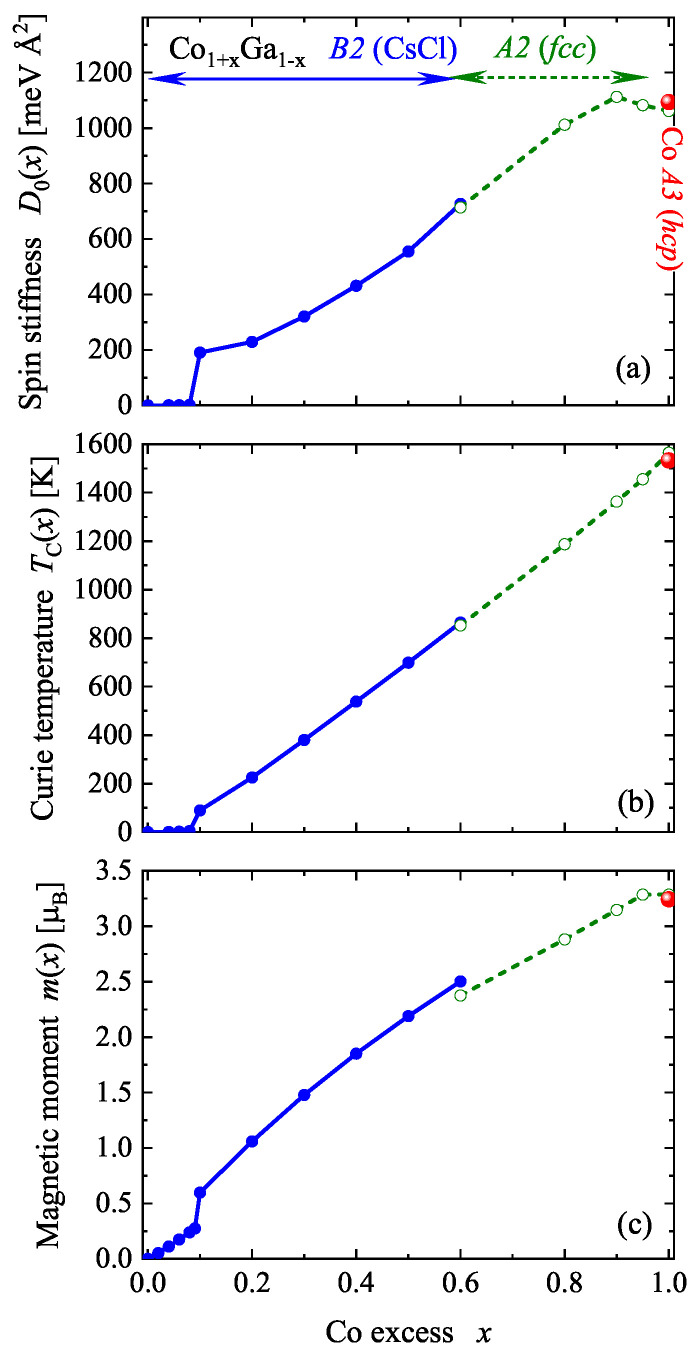
Magnetic properties of Co${}_{1+x}$Ga${}_{1-x}$ for 0≤x≤1. Shown are the spin stiffness (**a**), the Curie temperature (**b**) and the total magnetic moment (**c**). (Spin stiffness and total magnetic moment are given for a cell with two sites in all three cases.)

To complete the study, the situation at very high Co content is discussed. At Co content of above ≈70%, the structure of the alloy changes to the A1 fcc type. Indeed, mixtures of A1 and B2 ordered alloys may appear, depending on temperature during growth or measurements. Of note is the transition to the low-temperature hcp structure of Co i.e., the A3 type, above 95%. Calculations of the electronic and magnetic structure were performed for these high Co content alloys to complete the series. Finally, the behavior of the magnetic properties of the complete series Co${}_{1+x}$Ga${}_{1-x}$ for *x* from 0 to 1 is summarized in Figure 11.

The spin stiffness exhibits discontinuities at medium (small *x*) and very high Co content. In the ferromagnetic range, it increased nonlinearly from 200 to almost 1100 meVÅ${}^{2}$. Moreover, its behavior in the continuous region is different from that of the Curie temperature or magnetic moment. The Curie temperature exhibits a nearly linear dependence on the Co content from the onset of ferromagnetic order up to pure Co. It should be mentioned that the Curie temperatures for fcc and hcp Co are nearly identical (difficult to distinguish in Figure 11). At about 1550 K, the calculated mean field Curie temperature of Co is approximately 10% higher, compared to the experimental value of ≈1400 K. The mean field values of the high moment states in the vicinity of x=0.2 are similar to the experimental values reported in references [25,43,44]. Such deviations may be caused by partial disorder in the samples [45,46] that was not accounted for in the present calculations. It should be noted that large differences appear between the results of the various reported experiments. The magnetic moment increases above x=0.1 in a non-linear but nearly continuous manner. The magnetization does not follow the Slater–Pauling rule for localized ferromagnetic systems, but is on the borderline when its behavior is compared to that of itinerant ferromagnets [47,48]. However, the Curie temperature behaves similarly to that of the Co${}_{2}$-based Heusler compounds, even though the binary Co-Ga system is not half-metallic [49]. As mentioned above in Section 2.4, a small moment is observed for x≤0.08. In this compositional range, the Curie temperature is very small (below 5 K) and changes discontinuously to 90 K at x=0.1.

The behavior of the calculated magnetic properties agrees well with the results of reported experiments [14,23,44], in particular, for x<0.1 where the onset of ferromagnetism was observed at x≈0.07. The reported spin glass type property at lower Co concentration might be related to the localized magnetic moment of the excess Co atoms, due to the replacement of Ga on the 1a position.

## 3. Summary and Conclusions

In the present work, the electronic and magnetic structure of the Co-Ga alloy system was investigated. In particular, first principles calculations were performed for high Co content alloys which included Co${}_{1+x}$Ga${}_{1-x}$ with x≥0. The main emphasis was on the β phase where Co${}_{1+x}$Ga${}_{1-x}$ adopts a simple cubic CsCl type structure.

It was determined that well-ordered β-CoGa with 1:1 stoichiometry does not exhibit collective magnetism, but is a paramagnetic, metallic compound with a stable B2 structure. Calculations performed for slightly off-stoichiometric as well as stoichiometric CoGa revealed that neither Co nor Ga deficiency induced a magnetic order. In contrast to the types of disorder associated with Schottky type defects, ferromagnetism was found for Co-Ga anti-site disorder. A spin magnetic moment of about 1.2 $\mu_{B}$ was obtained for the completely disordered body-centered cubic structure.

As the Co content was increased, β-Co${}_{1+x}$Ga${}_{1-x}$ also became ferromagnetic when the excess Co replaced Ga. For an excess of Co of ≈10%, a discontinuity of the magnetic order was observed. The increasing magnetic exchange splitting caused a sudden change of the electronic structure, which resulted in a transition from a low moment to a high moment state. Thus, at 10% Co excess, a discontinuity of the magnetic moment, the Curie temperature and also of the electric conductivity was observed. In the high moment state, the resistivity has a maximum at about 37% Co excess. The differences in the resistivities for currents which were parallel or perpendicular to the magnetization are in the order of percent.

It was also observed that irrespective of the structural phase transitions into fcc and hcp structures at very high Co content, the magnetic moments and Curie temperatures increased continuously until they arrived at their maximum values for pure Co.

The most interesting and important finding is the discontinuity in the electronic and magnetic structure of the β-phase. In contrast to a Hume–Rothery type behavior, the magnetic order (but not the crystal structure) changes abruptly when increasing the Co content and thus changing the valence electron concentration.

## Figures and Tables

**Table 1:** QTAIMS critical point analysis of CoGa. pg is the point group symmetry of the critical point, and *W* is the Wyckoff position including the multiplicity of the critical points in the simple cubic cell.

pg	Type	Position			*W*	Name
$O_{h}$	nucleus	0	0	0	1a	Ga
$O_{h}$	nucleus	1/2	1/2	1/2	1b	Co
$D_{4h}$	bond	1/2	1/2	0	3c	$b_{1}$
$D_{4h}$	bond	1/2	0	0	3d	$b_{2}$
$C_{3v}$	bond	0.2481	0.2481	0.2481	8g	$b_{3}$
$C_{2v}$	ring	0.0828	0.0828	1/2	12j	$r_{1}$
$C_{2v}$	ring	0.4011	0.4011	0	12i	$r_{2}$
$C_{2v}$	cage	1/2	0.2106	0	12h	*c*

## Data Availability

Data are available from the author on reasonable request.

## Appendix A. Calculational Details

The electronic and magnetic properties were calculated by using the full potential ab-initio programs Wien2k [50,51] and Munich spr-kkr [52,53]. In both schemes, the generalized gradient approximation of Perdew, Burke, and Enzerhoff [54] was used for parametrization of the exchange-correlation functional.

The electronic structure of stoichiometric, well-ordered β-CoGa was calculated by using the full-potential linear augmented plane wave (FPLAPW) method as implemented in Wien2k [50,51]. The atoms were placed on the 1b (Co) and 1a (Ga) Wyckoff positions of the B2 structure. Calculations were performed by using $R_{\mathrm{MT}}k_{\max}=8$ for the number of plane waves and 1771 irreducible *k*-points based on a 40×40×40 mesh. Spin-orbit interaction was included to preserve the spin-orbit splitting of the Ga 3d semi-core level. The relaxation of the volume resulted in a lattice parameter of $a_{opt}=2.8812$ Å, which agrees very well with reported experimental values (2.855–2.8832 Å) [55]. The charge density was analyzed by using Bader’s quantum theory of atoms in molecules [33] (QTAIM) by using the built-in routines of Wien2k as well as the Critic2 package of programs [34,35]. The basis for the QTAIM analysis is a partition of real space into atomic regions, which are called Bader basins. The border surfaces of these basins are determined by a zero-flux condition of the charge density distribution. Critical points are also found from the behavior of the charge density, examples are minima and maxima, or the bond critical points that appear as first-order saddle points on the border surfaces. Integration over the basins was performed by using the qtree algorithm (for more details see references [34,35]).

The equilibrium formation enthalpy $∆H_{\mathrm{CoGa}}$ of ordered CoGa is given by the total energy $E_{\mathrm{CoGa}}$ taken with respect to equivalent amounts of the Co and Ga constituents, each in their equilibrium crystal structures:

(A1) $$∆H_{\mathrm{CoGa}}=E_{\mathrm{CoGa}}-\left(E_{\mathrm{Co}}+E_{\mathrm{Ga}}\right).$$

Note that the equilibrium structures of hcp Co and orthorhombic α-Ga contain 2 and 4 atoms, respectively. Sometimes, ∆H is called formation or cohesive energy. Here, a value of $∆H_{\mathrm{CoGa}}=-0.521$ eV, corresponding to −50.3 kJ/mol, was found. This is close to the range of values (−32 to −41 kJ/mol) obtained for CoGa by experiments for temperatures between 298 to 1173 K (see reference [56]).

The product of pressure and volume (p·V) in the enthalpy ($H=E_{tot}+p\cdot V$) was neglected because the pressure is zero in the equilibrium structure at T=0. Furthermore, the atomization or cohesive energy is given with respect to the free atom total energies $E_{\mathrm{coh}}=E_{\mathrm{solid}}-\sum E_{\mathrm{atom}}$. It is related to the enthalpy of sublimation ($H_{\mathrm{subl}}$). The calculated value for CoGa is in the order of −830 kJ/mol. This corresponds roughly to the energy needed to transfer solid CoGa into gaseous Co and Ga.

The full-potential, fully relativistic spin-polarized Munich spr-kkr package of Ebert et al. [52,53] is based on the Kohn–Korringa–Rostocker (KKR) Greens function method and facilitates calculations in the coherent potential approximation (CPA) [57] to address random-site occupations. Here, CPA was used to account for partial vacancies as well as to calculate the disordered CoGa and the alloy with varying composition. It should be mentioned that the mean field character of conventional CPA tends to underestimate local effects, in particular when short-range ordering, clustering [58], or changes of the local symmetry [59] are present. In such cases one may use nonlocal CPA as described in Reference [60].

For the self-consistent field cycles, the convergence criterion of Munich spr-kkr was set to $10^{-5}$ and 464 *k*-points from a 22×22×22 mesh were used for integration. The full symmetry potential was expanded in spherical harmonics up to l=6. The Lloyd formula [61,62] was used to find the correct Fermi energy and the Ga 3d electrons were treated as semi-core states. An imaginary part of 2 mRy was added to the potential when calculating the density of states or the Bloch spectral function. This corresponds to a thermal broadening of about 300 K.

The results of the electronic structure calculations for stoichiometric, well-ordered β-CoGa are the same as those obtained with the FPLAPW scheme of Wien2k. By using Munich spr-kkr, the equilibrium lattice parameter for β-CoGa is $a_{opt}=2.8963$ Å, which is about 0.5% larger than the result from Wien2k. By using CPA, a linear dependence of the optimized lattice parameter on the composition parameter *x* was obtained for Co${}_{1+x}$Ga${}_{1-x}$; that is, it follows Veegard’s law. In the range 0≤x≤0.6 with ∆x=0.1, it was found to be a(x)=(2.8978−0.0545x) Å. The maximum change of a(x) at x=0.6 is about −1%. Such small differences do not much affect the electronic and magnetic properties. The optimized lattice parameter for Co${}_{0.8}$Ga${}_{0.2}$ with Cu-type fcc structure was $a_{fcc}=3.617$ Å, which is about 0.6% larger than the experimental value [55].

The magnetic phase transition temperatures were calculated from the Heisenberg exchange integrals $J_{ij}$, as described in detail by Liechtenstein et al. [63,64]. The cluster size was set to 4.7 times the lattice parameter, which resulted in 4028 ij combinations overall for the exchange coupling parameter $J_{ij}$ for each atom. The spin wave stiffness *D* is defined from a quadratic dependence of the spin wave energy ($E\left(q\right)\approx Dq^{2}$) at small wave vectors (q≈0). It is given by

(A2) $$D=\frac{2}{3}\sum_{i,j}\frac{c_{ij}J_{ij}}{m_{ij}}r_{ij}^{2}.$$

For substances with a single magnetic atom, one has i=0, $m_{0j}=m$ is the magnetic moment and $r_{0j}$ are the distances to all sites of the lattice containing the magnetic atom. In the most general situation of alloys, one has random-site occupations by more than one type of magnetic atoms. In such cases, the exchange integrals are weighted by the concentrations $c_{ij}$ of the atoms at the different sites and the geometric mean of the magnetic moments ($m_{i,j}=\sqrt{m_{i}m_{j}}$) is used. In practice, the sum in Equation (A2) hardly converges because of the quadratic dependence on the distances. Therefore, the spin wave stiffness was calculated from the exchange integrals by using the extrapolation scheme of Pajda et al. [65].

Finally, the calculation of the transport properties is based on the Kubo–Greenwood approach [53,66,67,68]. In the investigation, the number of *k*-points was doubled for the calculations of the density of states and the Curie temperatures. This was further increased to 197173 irreducible *k*-points for the calculations of the conductivity.
